# Ultrasensitive Lateral Flow Immunoassay for Fumonisin B1 Detection Using Highly Luminescent Aggregation-Induced Emission Microbeads

**DOI:** 10.3390/toxins15010079

**Published:** 2023-01-16

**Authors:** Ge Xu, Xiaojing Fan, Xirui Chen, Zilong Liu, Guoxin Chen, Xiaxia Wei, Xiangmin Li, Yuankui Leng, Yonghua Xiong, Xiaolin Huang

**Affiliations:** 1 State Key Laboratory of Food Science and Technology, School of Food Science and Technology, Nanchang University, Nanchang 330047, China; 2 School of Future Technology, Nanchang University, Nanchang 330047, China; 3 School of Food Science and Engineering, Hainan University, Haikou 570228, China

**Keywords:** aggregation-induced emission, fluorescent microbeads, lateral flow immunoassay, fumonisin B1

## Abstract

Lateral flow immunoassay (LFIA) based on fluorescent microbeads has attracted much attention for its use in rapid and accurate food safety monitoring. However, conventional fluorescent microbeads are limited by the aggregation-caused quenching effect of the loaded fluorophores, thus resulting in low signal intensity and insufficient sensitivity of fluorescent LFIA. In this study, a green-emitting fluorophore with an aggregation-induced emission (AIE) characteristic was encapsulated in polymer nanoparticles via an emulsification technique to form ultrabright fluorescent microbeads (denoted as AIEMBs). The prepared AIEMBs were then applied in a competitive LFIA (AIE-LFIA) as signal reporters for the rapid and highly sensitive screening of fumonisin B1 (FB1) in real corn samples. High sensitivity with a detection limit of 0.024 ng/mL for FB1 was achieved by the developed AIE-LFIA. Excellent selectivity, good accuracy, and high reliability of the AIE-LFIA were demonstrated, indicating a promising platform for FB1 screening.

## 1. Introduction

Fumonisin B1 (FB1) is a Fusarium verticillioides-derived mycotoxin that commonly contaminates corn and other agricultural products [1,2]. Given the high threat it poses to the health of humans and animals, the highly efficient monitoring of FB1 in foods and feedstuffs is of great importance [3,4].

FB1 can be analyzed by instrumental methods (e.g., high-performance liquid chromatography [5] and liquid chromatography-tandem mass spectrometry [LC-MS/MS] [6]) with good accuracy and high reliability. However, these methods are conducted by expensive equipment with complex procedures by skilled technicians and their use is greatly limited for on-site detection of FB1 [7,8]. Immunoassays, including enzyme-linked immunosorbent assay and lateral flow immunoassay (LFIA), are widely used for the rapid screening of FB1 [9,10]. Among them, LFIA exhibits tremendous advantages in terms of rapidity, simplicity, and cost-effectiveness, and is very suitable for on-site screening in resource-poor areas [11,12,13,14].

Fluorescent microbeads have been used as advanced reporters of LFIA, enabling superior analysis compared to conventional colloidal gold nanoparticles (AuNPs) [15]. Organic dyes like fluorescein isothiocyanate [16] and quantum dots [17] are commonly used to produce fluorescent microbeads, which provide enhanced sensitivity when applied in developing LFIA. However, these fluorophores suffer from aggregation-caused quenching (ACQ) effect, thus limiting the signal intensity of conventional fluorescent microbeads [18,19,20].

The signal intensity of conventional fluorescent nanoparticles, which is highly influenced by the concentrations of loading fluorophores [21], is the core parameter for their sensitive utilization as reporters in LFIA [22]. However, the loading amounts of conventional fluorophores are limited because of their ACQ effect, resulting in limited signal intensity of fluorescent microbeads and the insufficient sensitivity of LFIA [23,24,25]. Hence, a novel type of fluorophores with aggregation-induced emission (AIE) effect, also called AIEgens, have attracted enormous attention in producing ultrabright nanoprobes [26,27,28,29]. Zhang et al. first proved that AIEgen-loaded microbeads are better probes of sandwich-type LFIA compared with two commercial fluorescent microbeads. The AIE microbead probe is about 5-times brighter and provides about 10 times the sensitivity of LFIA for detecting *Escherichia coli* O157:H7 [30].

**Scheme 1:**
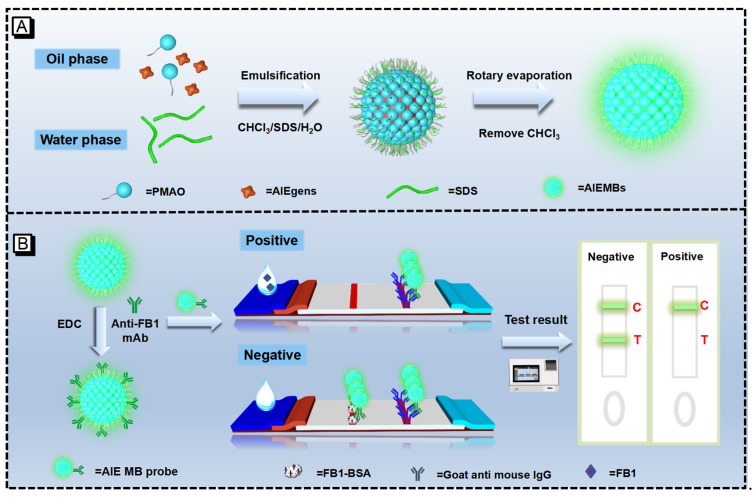
Schematic illustration of the AIE-LFIA for FB1 detection. (**A**) The synthetic route of the designed AIEMBs. (**B**) The diagram of AIE-LFIA for FB1 detection.

In this study, ultrabright AIE microbeads (AIEMBs) were adopted to develop a competitive LFIA for the rapid and highly sensitive detection of FB1 in corn. First, a green-emitting AIEgen was encapsulated in poly (maleic anhydride-alt-1-octadecene) (PMAO) via an emulsification method to prepare submicron-sized AIEMBs (Scheme 1A). High-density loading of AIEgen was achieved due to the deprivation of the ACQ effect, resulting in the ultrahigh brightness of AIEMBs. The prepared AIEMBs were then modified with anti-FB1 monoclonal antibodies (mAbs) and used as AIEMB probes in competing LFIA (denoted as AIE-LFIA) for the rapid sensing of FB1 (Scheme 1B). When FB1 is absent in the sample, the AIEMB probes are captured by the immobilized antigen (BSA-FB1) on the test (T) line and by goat anti-mouse IgG on the control (C) line, forming two bright-green bands on the test strips. By contrast, when FB1 is present, the binding of AIEMB probes with BSA-FB1 on the T line is inhibited, resulting in a darker or an invisible T line. Hence, the FB1 concentration can be quantified by the inhibition rate of 1-B/B_0_, where B_0_ and B represent the photoluminescent intensity ratio of the T line to C line (PI_T_/PI_C_), obtained from the detection of FB1-negative and FB1-positive samples, respectively.

## 2. Results and Discussion

### 2.1. Synthesis and Characterization of AIEMBs

The fluorophore used to prepare AIEMBs in this research was a green-emitting AIEgen. As shown in Figure S1, this AIEgen exhibits weak photoluminescence when dissolved in common organic solvents, while showing a dramatic increase in green emission with a peak of about 520 nm when water was added into the solvent. An enhancement of over 500 times in green emission was observed after an over 20% (*v*/*v*) water solution was added to the DMSO solution containing 1 mg/mL AIEgens.

The AIEgen was encapsulated in PMAO via an ultrasonic emulsification step to form O/W emulsions stabilized by Sodium dodecyl sulfonate (SDS), and submicron-sized AIEMBs formed after the chloroform in the oil droplets was evaporated. The composition of the oil phase, or the dosage ratio of AIEgen to PAMO, is crucial for preparing AIEMBs with high brightness and good stability. In theory, the loading of more AIEgens leads to higher brightness, while possibly weakening the structural stability or luminescent stability due to a thinner protective PMAO layer. As shown in Figure S2A,B, in our study the photoluminescent intensity of AIEMBs gradually increased with the increase of the dosage ratio of AIEgen to PMAO, from 1.5 to 4. The stability of the AIEMBs prepared from different dosage ratios of AIEgen to PMAO (1.5:1, 2:1, 3:1, 4:1) were then assessed. The results in Figure S2C indicate that these AIEMBs sprayed on the nitrocellulose (NC) membrane exhibited a decrease in photoluminescent intensity after 1 day of storage at 60 °C and an unchanged photoluminescent intensity in the subsequent 6 days. It is worth noting that more AIEgen loadings leads to a larger decrease in the photoluminescent intensity of the resultant AIEMBs during the first day of storage at 60 °C, indicating decreased stability due to a decreased thickness of the protective PMAO shell. Moreover, scanning electron microscope (SEM) image and hydrodynamic diameter distribution results showed that the higher loading of AIEgens results in a larger and wider size distribution of the AIEMBs (Figure S2D–K). Therefore, 2:1 was selected as the optimal dosage ratio of AIEgen to PMAO to prepare uniform AIEMBs with high brightness and good stability. In addition, the evaporation methods used to remove oil solvent are crucial to the solidification of the emulsions, thus affecting the morphology of resultant AIEMBs. Three evaporation methods including rotary evaporation at 40 °C, magnetic stirring at room temperature, and 60 °C incubation were investigated. SEM images and Dynamic light scattering (DLS) results, shown in Figure S3, show that rotary evaporation at 40 °C was the optimal evaporation method to achieve more uniform AIEMBs compared with the other two methods.

**Figure 1:**
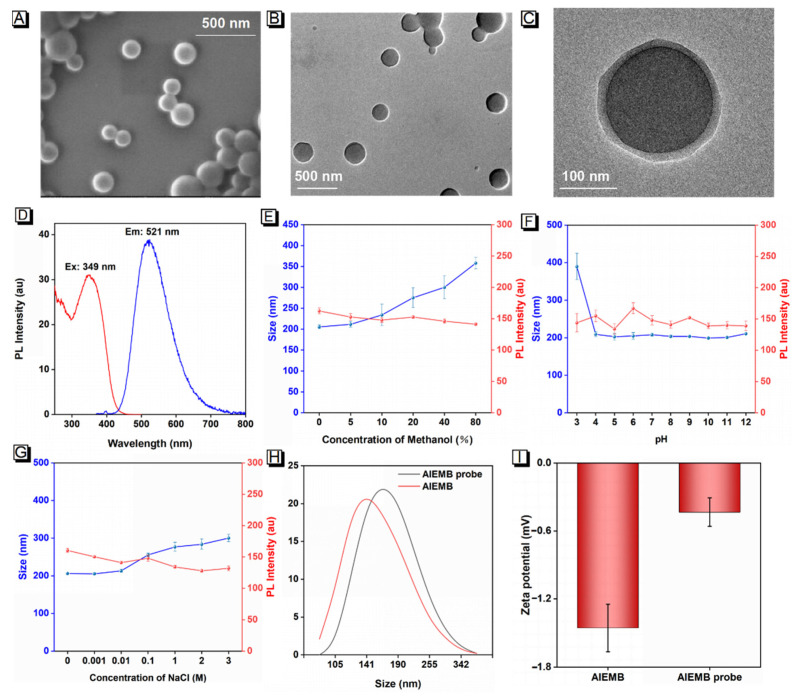
Characterization of AIEMBs and AIEMB probes. (**A**) SEM image. (**B**,**C**) TEM images. (**D**) Excitation (red line) and emission (blue line) spectra of AIEMBs. (**E**–**G**) The effect of methanol, ionic strength, and pH value on the size and photoluminescent intensity stability of AIEMBs (dosage ratio of AIEgens to PMAO = 2:1), which was assessed after 24 h of incubation in the corresponding aqueous solution. (**H**) DLS results and (**I**) Zeta-potential of AIEMBs and AIEMB probes. Vertical bars indicate the standard deviation (*n* = 3).

Under optimal synthetic conditions, the prepared AIEMBs exhibit a near-perfect sphere with a diameter of about 200 nm and a uniform size distribution (Figure 1A–C). The AIEMBs emit bright green photoluminescence with a maximum peak at 521 nm under the excitation wavelength of 350 nm (Figure 1D). The stability of the prepared AIEMBs against pH, ionic strength, and methanol (the organic solvent typically used for FB1 extraction) was then investigated. As shown in Figure 1E–G, no obvious fluctuations in the photoluminescent intensity of the AIEMBs were observed within 24 h of incubation in a buffer with wide ranges of methanol content (0–80%), pH (3–12), and NaCl concentration (0–3 M), while normally slight increases in the size of AIEMBs would be induced by high methanol content, high NaCl concentration and strong acidity, indicating a change in the hydration layer of the AIEMBs or slight aggregation. These results demonstrate an excellent fluorescent stability and acceptable colloidal stability of the AIEMBs in a buffer with wide ranges of methanol content, pH, and NaCl concentration. Hence, such highly bright and stable AIEMBs are good candidates for signal reporters for detecting real FB1 extract samples.

### 2.2. Development of AIE-LFIA

AIEMBs were used as signal reporters of an AIE-LFIA test strip with a competing antigen of BSA-FB1 conjugates sprayed as the T line and IgG sprayed as C line. The competitive binding of the AIEMB probe with the T line led to a negative correlation between the resultant PI_T_ and the FB1 concentration. The PI_C_ value signifies the validity of the AIE-LFIA test strip and was used as a reference to enable the accurate quantification of FB1.

Thereafter, anti-FB1 mAbs were immobilized onto the AIEMBs to prepare the AIEMB probes. The increase in average hydrodynamic diameter and the decrease in negative surface charge demonstrate the successful modification of the anti-FB1 mAbs on the surface of the AIEMBs (Figure 1H,I). To obtain AIEMB probes with a high affinity toward FB1, several key parameters for the anti-FB1 mAbs coupling, including pH value, 1-ethyl-3-(3-dimethylaminopropyl) carbodiimide (EDC) concentration, and labeling amounts of anti-FB1 mAbs, were optimized. AIEMB probes prepared with different conditions were evaluated by conducting AIE-LFIA for a FB1-negative sample and a FB1-positive sample (0.5 ng/mL), and PI_T_ value and the competition inhibition rate were adopted as screening indexes. A large PI_T_ value for FB1-negative samples indicating a good affinity and a high inhibition rate for FB1-negative samples indicating good competitive binding of FB1 toward AIEMB probes were expected. The results in Figure S4A–C demonstrate that the optimal AIEMB probes were prepared with 1 µg of EDC and 30 µg/mg (30 µg for 1 mg AIEMB) of anti-FB1 mAbs, in 0.01 M phosphate buffer (PB) solution with a pH value of 5.5.

The spraying density of the competing antigen of BSA-FB1 on the T line and the dosage of AIEMB probes are the two key factors affecting the competing binding process. Orthogonal AIE-LFIA experiments based on different dosages of AIEMB probes and BSA-FB1 concentrations in spraying solutions for the T line were conducted, and the PI_T_ value resulted from the FB1-negative sample and the competition inhibition rate resulting from the FB1-positive sample (0.35 ng/mL) were adopted as screening indexes. The results of the orthogonal experiments shown in Table S1 reveal that the optimal BSA-FB1 concentration in the spraying solution is 0.75 mg/mL and the optimal dosage of the AIEMB probe is 360 µg (5 µL, 72 μg/mL).

Changes in the pH and ionic strength of the sample solution could significantly influence the immunoreactions by altering the bioactivity of antigen-combining sites of the antibodies. A certain concentration of methanol in the extract solution which led to water-soluble protein degeneration was beneficial for eliminating matrix interference from real samples, while a high methanol content influenced the antigen–antibody interaction. Thus, the pH, NaCl concentration, and methanol concentration of the sample solution were successively optimized to achieve the best analytical performance (Figure S5). The PI_T_ resulting from detecting the FB1-negative sample by AIE-LFIA and the inhibition rate of a FB1-postive sample (0.35 ng/mL) were utilized to evaluate the effects of these parameters. The results obtained from detecting FB1-spiked PB (10 mM) samples with different pH values (Figure S5A) revealed that a high PI_T_ and the highest inhibition rate were achieved when the pH was 7.0. Then, the results obtained from detecting the FB1-spiked PB (10 mM, pH = 7.0) samples with different NaCl concentrations indicated that 50 mM NaCl is optimal for achieving the highest inhibition rate for the FB1-positive sample (Figure S5B). Similarly, 10% of methanol content was demonstrated to be optimal, according to the results from detecting FB1-spiked PBS (10 mM, pH = 7.0, 50 mM NaCl) samples with different methanol contents. Hence, the optimal sample solution is 0.01 M PBS buffer (pH = 7.0) containing 50 mM NaCl and 10% methanol, and the extracted corn samples should be diluted by 6-fold with PBS (10 mM, pH = 7.0, 50 mM NaCl) prior to AIE-LFIA analysis.

### 2.3. Analytical Performance of AIE-LFIA for FB1 Detection

**Figure 2:**
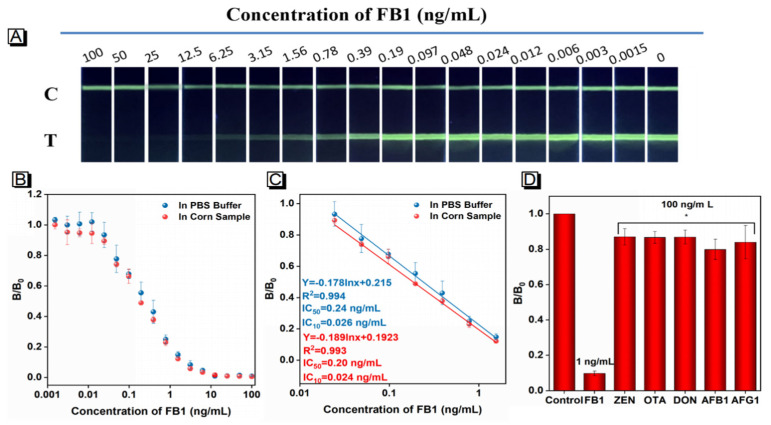
Analytical performance of the AIE-LFIA strips for FB1 detection under optimized conditions. (**A**) The resultant photographs of AIE-LFIA strips for a series of FB1-spiked PBS buffer (0.01 M, pH = 7.0, 50 mM NaCl, 10% methanol) samples. (**B**) The competitive inhibition curves of the AIE-LFIA for detecting FB1 in PBS buffer or corn extract solution. (**C**) The fitting competitive inhibition curves of AIE-LFIA strips within the linear detection range for detecting FB1 in PBS buffer or corn extract solution. (**D**) The selectivity of the AIE-LFIA method, assessed by detecting FB1 (1 ng/mL) and other common mycotoxins (100 ng/mL). Vertical bars indicate the standard deviation (*n* = 3).

Thereafter, the competitive inhibition curve of the proposed AIE-LFIA was obtained by detecting a series of FB1-spiked PBS (10 mM, pH = 7.0, 50 mM NaCl, 10% methanol) samples under optimal conditions (Figure 2A–C). The brightness of the T line gradually decreased with the increases of the FB1 concentration from 0 to 100 ng/mL, and the T line becomes invisible when the FB1 concentration increases to 3.15 ng/mL, which is defined as the naked-eye cut-off value. The AIE-LFIA demonstrates linear detection ranging from 0.024 to 1.56 ng/mL. The regression equation can be described as y = −0.178ln(x) + 0.215, and the half-maximum inhibitory concentration (IC_50_) and limit of detection (LOD, 10% inhibitory concentration [IC_10_]) were calculated to be 0.24 and 0.026 ng/mL, respectively.

By detecting FB1-spiked corn extract samples, prepared by the manual addition of FB1 in 6-fold diluted extract solution of FB1-negative corn with PBS (0.01 M, pH = 7.0, 50 mM NaCl), the matrix interference was evaluated. As shown in Figure 2B,C, the calibration curve of the established AIE-LFIA obtained from detecting FB1 in the corn extract is highly coincident with that from detecting FB1 in PBS (*p* > 0.05), indicating negligible matrix interference of real corn samples. The regression equation can be expressed as y = −0.189ln(x) + 0.1923, with the dynamic detection range from 0.024 ng/mL to 3.125 ng/mL. The IC_50_ and LOD are calculated as 0.20 ng/mL and 0.024 ng/mL, respectively, which are 3.7- and 9.2-fold lower than conventional AuNP based LIFA (AuNP-LFIA, Figure S7).

The selectivity of the proposed AIE-LFIA was then evaluated by detecting other corn-susceptible mycotoxins including zearalenone (ZEN), ochratoxin A (OTA), deoxynivalenol (DON), aflatoxin B1 (AFB1), and aflatoxin G1 (AFG1) at a high spiking concentration of 100 ng/mL. The results in Figure 2D demonstrate the excellent selectivity of the AIE-LFIA strip for FB1 detection. The accuracy and precision of this AIE-LFIA was evaluated by intra- and inter-assay for five FB1-spiked corn samples with FB1 concentrations of 5, 2.5, 1.25, 0.65, and 0.5 mg/kg. The average recoveries for the intra- and inter-assays range from 84.8% to 114.0% with coefficients of variation (CV) ranging from 4.05% to 7.82%, demonstrating an acceptable accuracy and precision for FB1 quantification (Table 1). Then, the reliability of the AIE-LFIA was validated by comparing results from detecting FB1-spiked corn samples with those of the LC-MS/MS method (Table 2). High correlation between the results of the two methods demonstrated the good reliability of our proposed AIE-LFIA. A literature survey in Table S2 also reveals that the proposed method exhibits superiority in sensitivity and time consumption, when compared with recently published methods for the rapid screening of FB1. In conclusion, the prepared green-emitting AIEMBs were better reporters of competing LFIA for FB1 sensing than AuNPs, providing enhanced sensitivity, good accuracy, and high reliability. Moreover, the use of AIEMBs can also be extended for competing LFIA for other chemical hazards.

## 3. Conclusions

In this study, an ultra-bright green-emitting AIEMB was successfully synthesized by encapsulating AIEgens in polymeric nanoparticles via an ultrasonic emulsification-rotary evaporation technique. By using the prepared AIEMBs as signal reporters, a highly sensitive competing AIE-LFIA for rapid FB1 screening was developed. A LOD of 0.024 ng/mL was attained for detecting FB1 in real corn samples, which is about 10-fold lower than conventional AuNP-LFIA. The proposed method also demonstrated, by inter- and intra-assays, satisfactory accuracy and precision for manually spiked corn samples. Good reliability for analyzing real corn samples was validated by a comparison with LC-MS/MS. In summary, this work proves the feasibility of using AIEMBs as signal probes to improve the sensitivity of competitive LFIA and provides a universal strategy for achieving rapid and accurate screening of small molecules like mycotoxins, pesticide residues, and other chemical hazards.

## 4. Materials and Methods

### 4.1. Materials and Reagents

AFB1, AFG1, ZEN, FB1, DON, and OTA were purchased from Huaan Magnech Bio-Tech Co., Ltd. (Beijing, China). SDS, bovine serum albumin (BSA), EDC, and PMAO (M_W_ = 30,000~50,000 Da) were obtained from Sigma-Aldrich (St. Louis, MO, USA). AIEgens with an excitation and emission wavelength of 350 nm and 520 nm were purchased from Jiangxi Weibang Biotechnology Co., Ltd. Anti-FB1 mAbs and BSA-FB1 conjugates were purchased from Jie Sheng Jie Kang Bio-Tech Co., Ltd. (Wuxi, China). The sample pad, absorbent pad, and NC membrane were provided by Wuxi Zodolabs Biotech Co., Ltd. (Wuxi, China). Goat anti-mouse IgG was obtained from Chongqing Xinyuanjiahe Biotechnology Inc. (Chongqing, China). Other chemicals were of analytical grade and purchased from Sinopharm Chemical Corp. (Shanghai, China). All reagents were used without further purification.

### 4.2. Characterization

The morphology of the prepared AIEMBs was investigated using a JEOL JEM 2100 transmission electron microscope (TEM, Tokyo, Japan). The size distribution of various AIEMBs was analyzed via DLS, performed on a Zetasizer Nano-ZEN3700 instrument (Malvern, UK). UV-Vis absorption spectra were obtained using an Amersham Pharmacia Ultrospec 4300 pro UV/visible spectrophotometer (England, UK). Fluorescence intensity of AIEMBs was monitored using a Hitachi F-4500 fluorescence spectrophotometer (Tokyo, Japan). The ultrasonic homogenizer was supplied by Ningbo Scientz Biotechnology Co., Ltd. (Ningbo, China). The Bio-Dot XYZ3000 automatic dot film tester was supplied by BioDot (Irvine, CA). The automatic programmable cutter was purchased from Shanghai Jinbiao Biotechnology (Shanghai, China). The fluorescence reader (model number: FIC-Q1) was custom-made by Huguo Science Instrument Co., Ltd. (Shanghai, China). FB1 residues in real corn samples were confirmed by an LC-MS/MS instrument (Agilengt 1290-6538, Palo Alto, CA, USA).

### 4.3. Synthesis of AIEMBs and AIEMB Probes

The AIEMBs were prepared via an emulsification-solvent evaporation process. In brief, 50 µL of chloroform solution containing certain amounts of AIEgens and PMAO were added into 500 µL of aqueous solution containing SDS (12 mg/mL). The mixture was emulsified for 3 min in an ice bath by using an ultrasonic processor at a power of 120 W. Subsequently, the chloroform in the O/W emulsion droplets was removed through rotary evaporation at 40 °C, and then the AIEMBs were collected via centrifugation (13,500 rpm, 15 min) at 4 °C. The pellet was resuspended in 0.01 M PB buffer (pH = 10) for 24 h to hydrolyze the anhydride groups of PMAO into the carboxyl groups. Finally, the resultant carboxylic AIEMBs were collected and washed three times with water via centrifugation.

The AIEMB probes were prepared by immobilizing anti-FB1 mAbs onto the surface of AIEMBs via the carbodiimide method. Typically, 0.216 μg of anti-FB1 mAbs and 7.2 µg of AIEMBs were mixed in 500 µL of PB solution (0.01 M, pH = 5.5), and a freshly prepared PB solution containing 1 µg of EDC was added. After an incubation of 90 min at room temperature, 10 mg of BSA were added for another 1 h of incubation. The resultant AIEMB probes were collected via centrifugation and resuspended in 100 µL of PB (0.01 M, pH = 7.4) containing 25 wt.% sucrose, 1 wt.% BSA, and 0.1 wt.% sodiumazide.

### 4.4. Construction of the AIE-LFIA Test Strips

The AIE-LFIA test strips were constructed according to our previous report with slight modifications. A total of 0.75 mg/mL of BSA-FB1 conjugates and 0.5 mg/mL of goat anti-mouse IgG were sprayed onto the NC membrane as the T and C lines at a density of 0.6 mL/cm using a ZX-1000 dispensing platform. The distance between the two lines was 6.0 mm. The NC membrane was then vacuum-dried overnight at 37 °C. Then, the sample pad, NC membrane, and adsorbent pad were laminated, attached onto the polyvinyl chloride (PVC) baseplate, and the assembled strip was cut into 3.9 mm-wide test strips. The test strips were inserted into rigid plastic cassettes with a sample well and a reading window. The prepared test strips were sealed in a plastic bag with desiccant gel for further use.

### 4.5. Detection Procedure of AIE-LFIA for FB1

3 µL of solution containing AIEMB probes (72 μg/mL) were incubated with 70 µL of sample solution for 5 min, and the mixed solution was then added to the sample well of the test strip. After 15 min, the photoluminescent intensities at the T and C lines (PI_T_ and PI_C_) were recorded using a commercial fluorescence strip reader. Finally, the FB1 concentration was calculated based on the competitive inhibition curve and the resultant inhibition rate of 1-B/B_0_.

### 4.6. Sample Preparation

Real corn samples confirmed to be FB1-free by the LC-MS/MS method were purchased from a local supermarket. The corn powder was manually spiked with FB1 and extracted according to our previously reported method [9]. Briefly, 4.0 g of FB1-contaminated pulverized corn sample were extracted with 4 mL extractant of methanol-water (60:40, *v/v*) for 20 min on a vortex shaker. The mixture was centrifuged at 6000 rpm for 15 min, and then the supernatant was stored as an extracted sample at −20 °C. Prior to AIE-LFIA detection, the extracted sample was diluted by 6-fold with PBS (0.01 M, pH = 7.4, 50 mM NaCl).

## Figures and Tables

**Table 1:** Precision and accuracy of the AIE-LFIA for FB1-spiked corn samples.

FB1-Spiked Concentration (mg/kg)	Intra-Assay (*n* = 3)			Inter-Assay (*n* = 3)		
	Recovered Concentration (mg/kg)	Recovery (%)	CV (%)	Recovered Concentration (mg/kg)	Recovery (%)	CV (%)
5.00	4.98	99.6	4.05	5.35	107.0	6.33
2.50	2.63	105.2	5.52	2.67	106.8	7.82
1.25	1.09	87.2	4.12	1.06	84.8	4.98
0.63	0.59	93.7	4.85	0.58	92.1	7.67
0.50	0.57	114.0	7.12	0.57	114.0	6.60

**Note:** Repeatability and reproducibility were evaluated by the addition and recovery experiment with intra- and inter-assay, in which intra-assay was carried out in triplicate on the same day and intra-assay was performed on three consecutive days [9].

**Table 2:** Comparison of the developed AIEMB-LFIA and LC-MS/MS.

Spiked Concentration (mg/kg)	AIE-LFIA		LC-MS/MS	
	Recovered Concentration (mg/kg)	CV (%)	Recovered Concentration (mg/kg)	CV (%)
5.00	5.19	7.49	5.54	5.34
2.50	2.66	9.52	2.85	6.89
1.25	1.10	8.12	1.45	4.98
0.63	0.60	6.87	0.77	6.88
0.50	0.57	8.99	0.54	6.45

## Data Availability

Not applicable.

## Supplementary Materials

The following supporting information can be downloaded at: https://www.mdpi.com/article/10.3390/toxins15010079/s1: Figure S1: Characterizations of AIEgens. (A) Photoluminescence spectrum of AIEgens in different organic solvents, including methanol (MeOH), ethanol (EtOH), acetonitrile (ACN), dimethylformamide (DMF), dimethyl sulfoxide (DMSO), tetrahydrofuran (THF), dichloromethane (DCM), and trichloromethane (TCM). (B) Photoluminescent intensity and (C) enhanced factor in photoluminescent intensity of AIEgens in DMSO/water mixtures with different water fractions compared with that in pure DMSO (i.e., P0); Figure S2: Optimization of dosage ratio of AIEgens to PMAO for preparing AIEMBs (A) The relationship between the photoluminescent intensity of resultant AIEMB solutions and the dosage of AIEgens, with 5 mg of PMAO dosage. (B) The photoluminescence spectra of resultant AIEMB solutions, with 5 mg of PMAO dosage. (C) Photoluminescence stability of AIEMBs synthesized with different AIEgen dosage at 60 ℃, assessed by P/P0, where P0 and P represent photoluminescent intensity of AIEMB sprayed on NC membrane before and after 1–7 d storage at 60 ℃. (D–K) The SEM images and DLS results of AIEgens synthesized from different dosage ratios of AIEgen to PMAO. (D,H) 1.5:1; (E,I) 2:1; (F,J) 3:1; (G,K) 4:1; Figure S3: The effect of condition for chloroform evaporation on the resultant AIEMBs. SEM images and DLS results of the AIEMBs formed after different chloroform evaporation processes: (A,E) vibrating at 60 ℃, (B,F) magnetic stirring at room temperature and (C,G) rotary evaporation at 40 ℃ to remove CHCl3; Figure S4: Optimization of the (A) pH value, (B) Dosage of EDC, and (C) Labeling amounts of antibodies in a 500 μL solution containing AIEMBs for preparing AIEMB probes, according to the analytical performance when used in AIE-LFIA test strips for FB1 detection. Vertical bars indicate the standard deviation (*n* = 3); Table S1: Optimization of the concentration of FB1-BSA for spraying T line and dosage of AIEMB probes via an orthogonal experiment; Figure S5. The optimization of sample solution. Effects of the pH value(A), concentration of NaCl (B), and methanol concentration (C) in the sample solution on the resultant PI_T_ value for FB1-negative sample and competitive inhibition rates for the FB1-positive sample (0.35 ng/mL). Vertical bars indicate the standard deviation (*n* = 3); Figure S6: Optimization of preparation of AuNP probes. The effects of (A) amount of K_2_CO_3_ (pH controlling agent) and (B) Labeling amounts of antibodies in a 500 μL solution containing 1.17 nM AuNPs on the performance of resultant AuNP probes, assessed by a AuNP-LFIA for FB1 detection. OD_T_ represents optical density of T line, vertical bars indicate the standard deviation (*n* = 3); Figure S7: (A) The competitive inhibition curves of the conventional AuNP-LFIA for detecting FB1 in corn extract solution. (B) The fitting competitive inhibition curves of conventional AuNP-LFIA within linear detection range for detecting FB1 in corn extract solution. where B0 and B represent optical intensity ratio of T line to C line (OD_T_/OD_C_) resulted from detecting FB1-negative and FB1-positive samples, respectively. Vertical bars indicate the standard deviation (*n* = 3); Table S2: A summary of recently reported FB1 analysis platforms. References see [9,31,32,33,34,35,36,37,38].
