# Supplementary material for: Ultrasensitive Lateral Flow Immunoassay for Fumonisin B1 Detection Using Highly Luminescent Aggregation-Induced Emission Microbeads
Source: Toxins (Basel). 2023 Jan 16;15(1):79. doi: 10.3390/toxins15010079 (PMC9861643; doi:10.3390/toxins15010079)
Supplement: Supplementary file 1 [file toxins-15-00079-s001.zip › toxins-2113726-supplementary.pdf]

Supplementary Information for:

Ultrasensitive lateral flow immunoassay for fumonisin B1 detection using highly luminescent aggregation-induced emission microbeads

Ge Xu [1,†], Xiaojing Fan [2,†], Xirui Chen [1], Zilong Liu [3], Guoxin Chen [1], Xiaxia Wei [1], Xiangmin Li [1], Yuankui Leng [1,*], Yonghua Xiong [1] and Xiaolin Huang [1,*]

[1] State Key Laboratory of Food Science and Technology, School of Food Science and Technology, Nanchang University, Nanchang 330047, China; xuge@email.ncu.edu.cn (G.X.); cxirui219921@163.com (X.C.); 402313320031@email.ncu.edu.cn (G.C.); 412326520009@email.ncu.edu.cn (X.W.); lixiangmin73@163.com (X.L.); xiongyonghua@ncu.edu.cn (Y.X.)

[2] School of Future Technology, Nanchang University, Nanchang 330047, China; 5701121069@email.ncu.edu.cn

[3] School of Food Science and Engineering, Hainan University, Haikou 570228, China; zilongliu@hainanu.edu.cn

† These authors contributed equally to this work.

* Correspondence: ykleng@ncu.edu.cn (Y.L.); xiaolin.huang@ncu.edu.cn (X.H.)

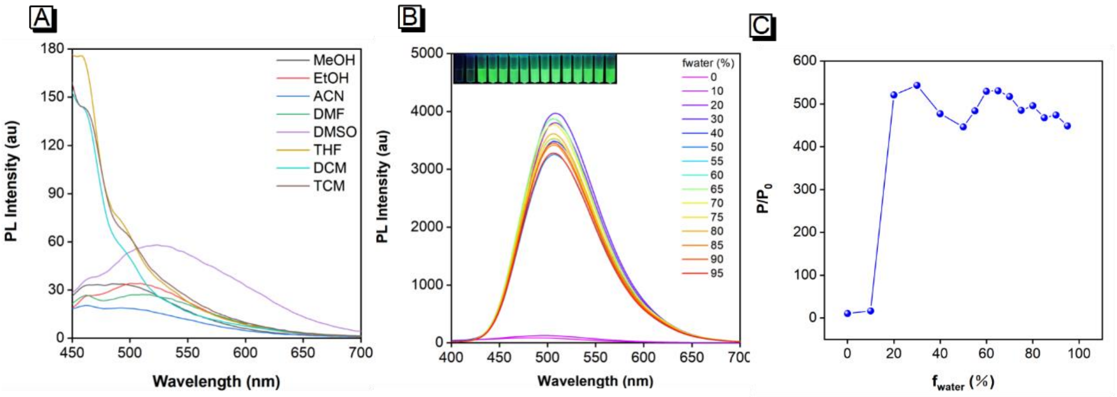

Supplementary Figure S1. Characterizations of AIEgens. (A) Photoluminescence spectrum of AIEgens in different organic solvents, including methanol (MeOH), ethanol (EtOH), acetonitrile (ACN), dimethylformamide (DMF), dimethyl sulfoxide (DMSO), tetrahydrofuran (THF), dichloromethane (DCM), and trichloromethane (TCM). (B) Photoluminescent intensity and (C) enhanced factor in photoluminescent intensity of AIEgens in DMSO/water mixtures with different water fractions compared with that in pure DMSO (i.e., P_0).

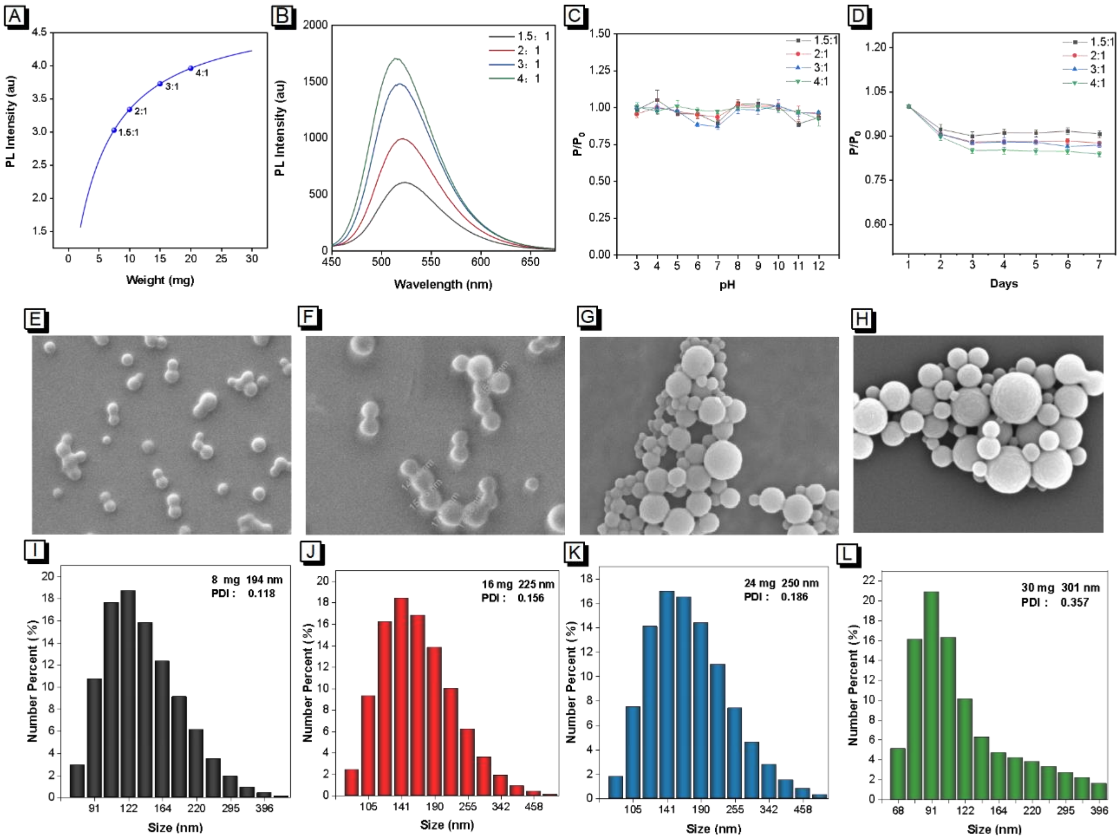

Supplementary Figure S2. Optimization of dosage ratio of AIEgens to PMAO for preparing AIEMBs (A) The relationship between the photoluminescent intensity of resultant AIEMB solutions and the dosage of AIEgens, with 5mg of PMAO dosage. (B) The photoluminescence spectra of resultant AIEMB solutions, with 5 mg of PMAO dosage. (C) Photoluminescence stability of AIEMBs synthesized with different AIEgen dosage at 60 ℃, assessed by P/P_0, where P_0 and P represent photoluminescent intensity of AIEMB sprayed on NC membrane before and after 1-7 d storage at 60 ℃. (D-K) The SEM images and DLS results of AIEgens synthesized from different dosage ratios of AIEgen to PMAO. (D, H) 1.5:1; (E, I) 2:1; (F, J) 3:1; (G, K) 4:1.

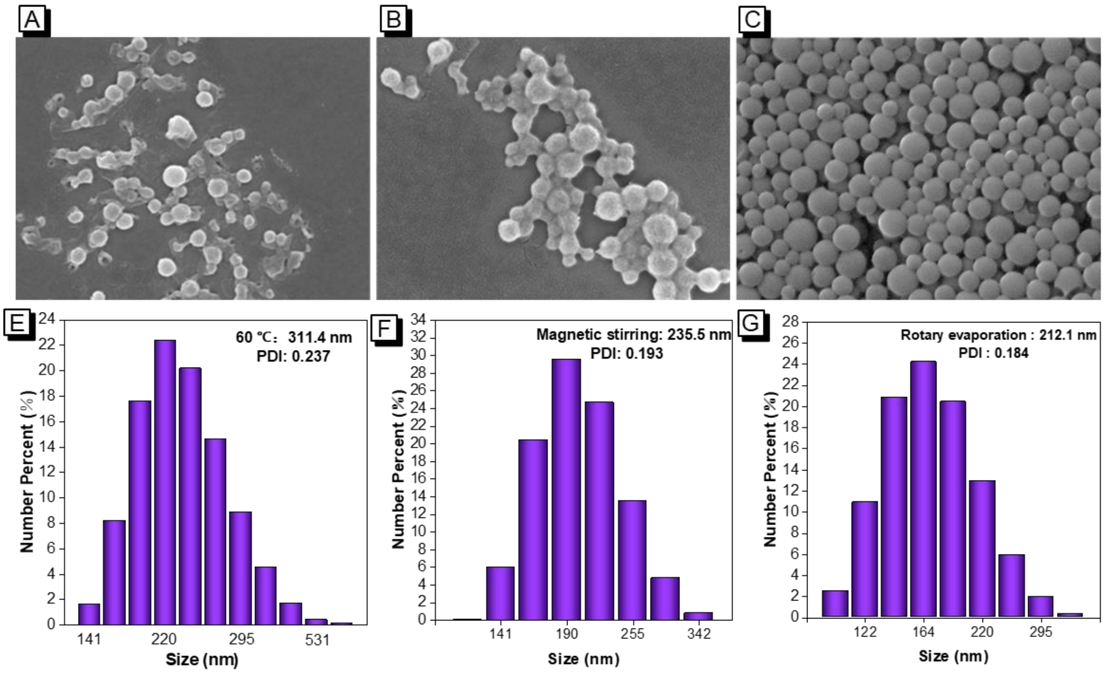

Supplementary Figure S3. The effect of condition for chloroform evaporation on the resultant AIEMBs. SEM images and DLS results of the AIEMBs formed after different chloroform evaporation processes: (A, E) vibrating at 60 ℃, (B, F) magnetic stirring at room temperature and (C, G) rotary evaporation at 40 ℃ to remove $CHCl_3$.

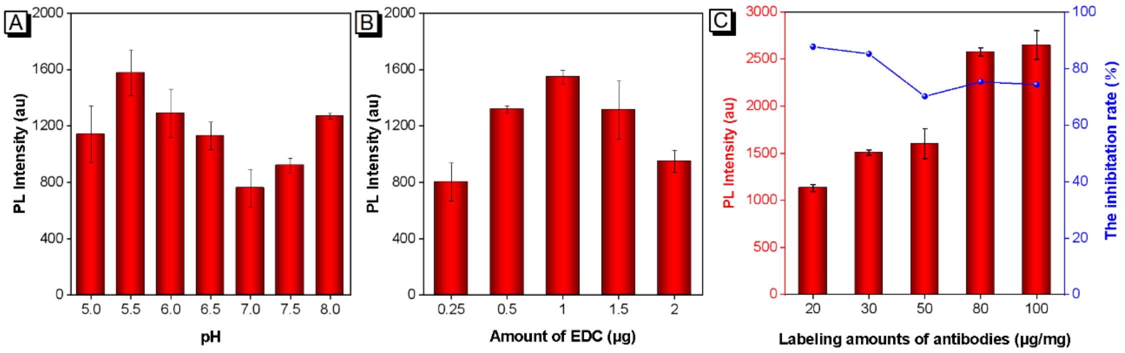

Supplementary Figure S4. Optimization of the (A) pH value, (B) Dosage of EDC, and (C) Labeling amounts of antibodies in a 500 μL solution containing AIEMBs for preparing AIEMB probes, according to the analytical performance when used in AIE-LFIA test strips for FB1 detection. Vertical bars indicate the standard deviation (n = 3).

Supplementary Table S1. Optimization of the concentration of FB1-BSA for spraying T line and dosage of AIEMB probes via an orthogonal experiment.

The concentration of FB1-BSA (mg/mL)	The volume of AIEMB probes (μL) [c]	PI_T (negative)	PI_T/PI_C (positive)	The inhibition rate [a]
1	3	8816 ± 472	3.26 ± 0.361	0.69 ± 0.004
1	5	18521 ± 732	3.61 ± 0.238	0.65 ± 0.009
1	7	24216 ± 684	2.77 ± 0.163	0.66 ± 0.023
0.75	3	4547 ± 429	1.32 ± 0.109	0.71 ± 0.016
0.75 [b]	5	8586 ± 482	1.27 ± 0.044	0.70 ± 0.045
0.75	7	12943 ± 116	1.30 ± 0.085	0.65 ± 0.045
0.5	3	3624 ± 348	2.25 ± 0.302	0.77 ± 0.006
0.5	5	8856 ± 306	1.17 ± 0.033	0.66 ± 0.033
0.5	7	12804 ± 451	1.44 ± 0.019	0.64 ± 0.012

a Inhibition rates are obtained from detecting a FB1-positive sample (0.35 ng/mL).
b Optimal parameters of AIE-LFIA.
c The concentration of AIEMB probes is 72 μg/mL.

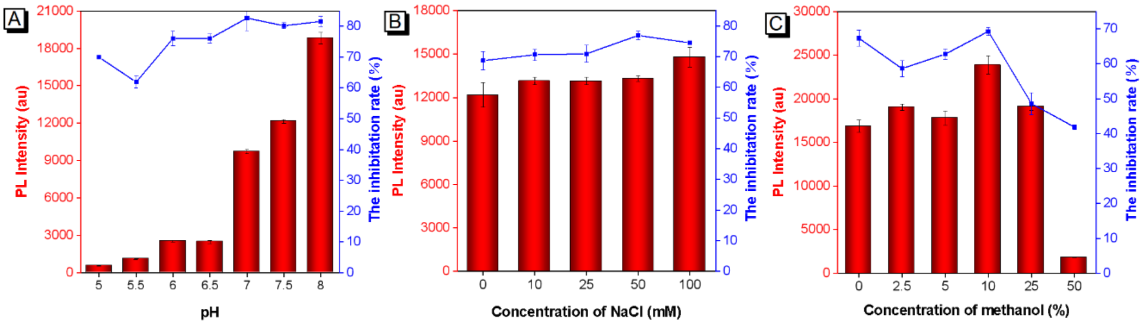

Supplementary Figure S5. The optimization of sample solution. Effects of the pH value(A), concentration of NaCl (B), and methanol concentration (C) in the sample solution on the resultant PI_T value for FB1-negative sample and competitive inhibition rates for the FB1-positive sample (0.35 ng/mL). Vertical bars indicate the standard deviation (n = 3).

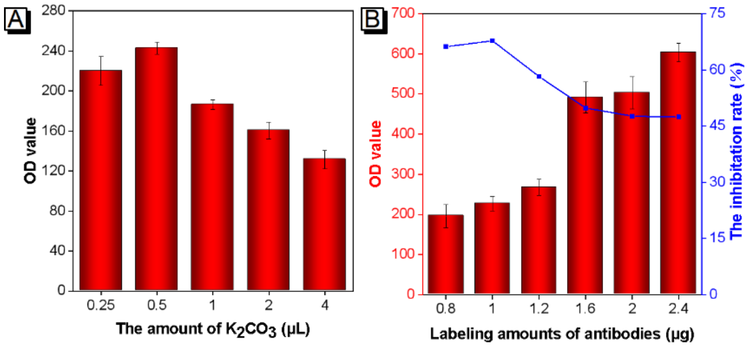

Supplementary Figure S6. Optimization of preparation of AuNP probes. The effects of (A) amount of K2CO3 (pH controlling agent) and (B) Labeling amounts of antibodies in a 500 μL solution containing 1.17 nM AuNPs on the performance of resultant AuNP probes, assessed by a AuNP-LFIA for FB1 detection. OD_T represents optical density of T line, vertical bars indicate the standard deviation (n = 3).

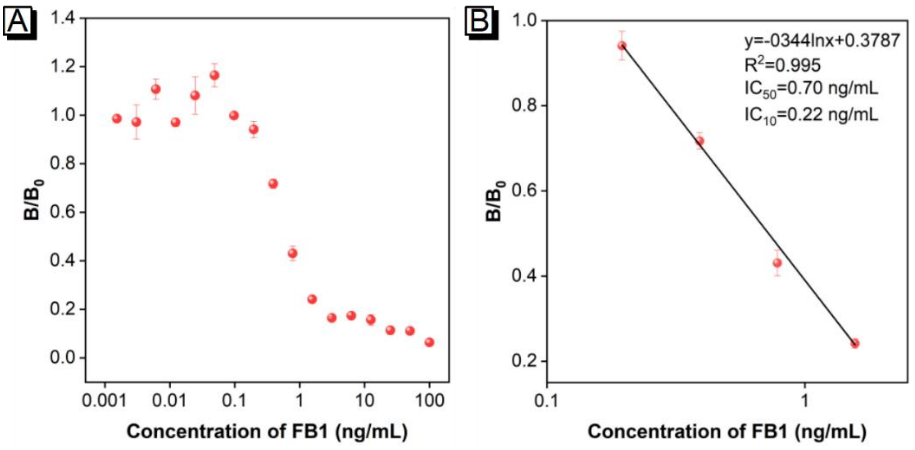

Supplementary Figure S7. (A) The competitive inhibition curves of the conventional AuNP-LFIA for detecting FB1 in corn extract solution. (B) The fitting competitive inhibition curves of conventional AuNP-LFIA within linear detection range for detecting FB1 in corn extract solution. where B0 and B represent optical intensity ratio of T line to C line (ODT/ODC) resulted from detecting FB1-negative and FB1-positive samples, respectively. Vertical bars indicate the standard deviation (n = 3).

Supplementary Table S2. A summary of recently reported FB1 analysis platforms.

Reported Method	LOD (ng/mL)	Detection time (min)	Refs
LFIA based on time-resolved luminescence nanoparticles	0.025	15	1
Quantum dot nanobead-based LFIA	2.29	15	2
Quantum dot nanobead-based LFIA	1.58	15	3
LFIA using gold nanobeads as reporters	1.76	15	4
LFIA using colloidal gold nanoparticles as reporters	2.5	15	5
Competitive HRP-Linked colorimetric aptasensor	0.3	>60	6
Gold growth-based plasmonic ELISA	0.31	60	7
Competitive plasmonic ELISA based on catalyzed aggregation of gold nanoparticle	3.07	60	8
Competitive fluorescence ELISA using CdTe quantum dots as reporters	0.33	>60	9
AIEMBs	0.02	15	This work

References

1. Zha, C.; An, X.; Zhang, J.; Wei, L.; Zhang, Q.; Yang, Q.; Li, F.; Sun, X.; Guo, Y., Indirect signal amplification strategy with a universal probe-based lateral flow immunoassay for the rapid quantitative detection of fumonisin B1. Anal Methods **2022**, 14 (7), 708-716.
2. Hou, S.; Ma, J.; Cheng, Y.; Wang, H.; Sun, J.; Yan, Y., Quantum dot nanobead-based fluorescent immunochromatographic assay for simultaneous quantitative detection of fumonisin B1, dexyonivalenol, and zearalenone in grains. Food Control **2020**, 117, 107331.
3. Shao, Y.; Duan, H.; Zhou, S.; Ma, T.; Guo, L.; Huang, X.; Xiong, Y., Biotin-streptavidin system-mediated ratiometric multiplex immunochromatographic assay for simultaneous and accurate quantification of three mycotoxins. J Agric Food Chem **2019**, 67 (32), 9022-9031.
4. Chen, X.; Miao, X.; Ma, T.; Leng, Y.; Hao, L.; Duan, H.; Yuan, J.; Li, Y.; Huang, X.; Xiong, Y., Gold nanobeads with enhanced absorbance for improved sensitivity in competitive lateral flow immunoassays. Foods **2021**, 10 (7), 1488.
5. Ren, W.; Xu, Y.; Huang, Z.; Li, Y.; Tu, Z.; Zou, L.; He, Q.; Fu, J.; Liu, S.; Hammock, B. D., Single-chain variable fragment antibody-based immunochromatographic strip for rapid detection of fumonisin B1 in maize samples. Food Chem **2020**, 319, 126546.
6. Tao, Z.; Zhou, Y.; Li, X.; Wang, Z., Competitive HRP-linked colorimetric aptasensor for the setection of fumonisin B1 in food based on dual biotin-streptavidin interaction. Biosensors (Basel) **2020**, 10 (4), 31.
7. Zhan, S.; Zheng, L.; Zhou, Y.; Wu, K.; Duan, H.; Huang, X.; Xiong, Y., A gold growth-based plasmonic ELISA for the sensitive detection of fumonisin B1 in maize. Toxins (Basel) **2019**, 11 (6), 323.
8. Chen, X.; Liang, Y.; Zhang, W.; Leng, Y.; Xiong, Y., A colorimetric immunoassay based on glucose oxidase-induced AuNP aggregation for the detection of fumonisin B1. Talanta **2018**, 186, 29-35.
9. Lu, T.; Zhan, S.; Zhou, Y.; Chen, X.; Huang, X.; Leng, Y.; Xiong, Y.; Xu, Y., Fluorescence ELISA based on CAT-regulated fluorescence quenching of CdTe QDs for sensitive detection of FB1. Anal Methods **2018**, 10 (48), 5797-5802.
